# Exploring the Potential of Cellulose Nanocrystals Originated from Ramie (*Boehmeria nivea* L. Gaud) in Formation of Microspheres for Enhanced Solubility of Furosemide

**DOI:** 10.3390/polym17131879

**Published:** 2025-07-05

**Authors:** Anis Yohana Chaerunisaa, Yoga Windhu Wardhana, Mayang Kusuma Dewi, Margaretha Efa Putri, Fitriani Jati Rahmania

**Affiliations:** 1 Department of Pharmaceutics and Pharmaceutical Technology, Faculty of Pharmacy, Universitas Padjadjaran, Sumedang 45363, Indonesia; y.w.wardhana@unpad.ac.id (Y.W.W.); mayang14001@mail.unpad.ac.id (M.K.D.); 2 Dosage Form Development Research Centre, Faculty of Pharmacy, Universitas Padjadjaran, Sumedang 45363, Indonesia; 3 Magister Program, Faculty of Pharmacy, Universitas Padjadjaran, Sumedang 45363, Indonesia; margaretha17003@mail.unpad.ac.id; 4 Faculty of Pharmacy, Universitas Padjadjaran, Sumedang 45363, Indonesia; fitriani.jr@itb.ac.id; 5 Graduate Institute of Applied Science and Technology, National Taiwan University of Science and Technology, Taipei 10607, Taiwan

**Keywords:** *Boehmeria nivea* L. Gaud., furosemide, microspheres, nanocrystalline cellulose, ramie

## Abstract

Cellulose nanocrystals possess unique properties such as high surface area and excellent biocompatibility. They can disrupt strong hydrogen bonds and other intermolecular forces that hinder the solubility of certain molecules thus enhancing the solubility of poorly soluble materials. The main challenge in formulating poorly soluble drugs lies in their limited therapeutic efficacy due to inadequate solubility and bioavailability. Therefore, an innovative approach such as using cellulose nanocrystals to enhance the solubility is highly needed. The aim of this research is to study the potential of ramie (*Boehmeria nivea* L. Gaud) as a source of cellulose nanocrystals in the development of microspheres for the solubility enhancement of poorly soluble drugs. Nanocrystalline cellulose was isolated from the ramie (*Boehmeria nivea* L. Gaud) by optimizing hydrolysis conditions with varying acid concentrations and reaction times. Characterizations were performed by measuring particle size, pH, and sulfate content, followed by morphological study by SEM, functional group analysis, and thermal analysis. The use of sulfuric acid in the hydrolysis process of flax cellulose at 45 °C, as the type of acid that gives the best results, at 50% acid concentration for 60 min produces cellulose nanocrystallines with a particle size of 120 nm, sulfate concentration density of 133.09 mmol/kg, crystallinity of 96.2%, and a yield of 63.24 ± 8.72%. Furosemide was used as the poorly soluble drug model and its solubility enhancement in the form of furosemide/RNCC microspheres was evaluated through saturated solubility testing and in vitro dissolution. This study demonstrated that RNCC could improve the solubility of furosemide, which contributes to developing sustainable drug formulations and eco-friendly delivery systems for poorly soluble drugs.

## 1. Introduction

The field of pharmaceutical research is continuously seeking innovative strategies to enhance drug solubility and bioavailability, especially for poorly water-soluble drugs like furosemide, a widely used diuretic for treating hypertension and edema that belongs to Biopharmaceutical Classification System (BCS) class IV, which hinders its optimal therapeutic delivery [1,2]. In recent years, nanostructures obtained from plant-based sources such as starch and cellulose have gained attention due to their cost-effectiveness, sustainability, and renewability. These materials exhibit exceptional physicochemical properties, making them well-suited for a diverse range of applications [3].

Cellulose has demonstrated versatile applications as a drug-delivery vehicle [4,5]. Microcrystalline cellulose (MCC), a common form of cellulose, serves as a multifunctional component in tablet formulations, acting as a filler, binder, disintegrant, and crushing agent [6,7]. Despite its advantageous disintegrating properties attributed to its hydrophilic nature and high water absorption, MCC exhibits limitations, including a lower crushing strength compared to other super disintegrant classes [6].

Addressing the challenges associated with drug solubility, physical methods such as particle size reduction and the formation of nanocrystals are often used [8,9]. Additionally, nanoparticles can enhance drug diffusion by forming colloids in the nanoparticle system to increase drug concentration on the intestinal membrane for absorption [10]. Therefore, the physical use of nanocrystalline cellulose (NCC) can improve drug solubility better than MCC [11]. NCC has gained attention for its potential to improve the solubility of BCS class II drugs such as ibuprofen derivatives, nifedipine, and indomethacin by increasing their solubility [12,13,14]. The unique properties of NCC, including mucoadhesive characteristics and interaction with phospholipids on the intestinal cell membrane, make it a promising carrier for enhancing drug bioavailability, especially for BCS class IV substances [15,16].

NCC can be derived from various agro-waste sources, such as date palms [17], wood [18], jackfruit [19], cucumber [20], seaweed [21], cotton fiber [22], and ramie [23]. Among those sources, ramie has been cultivated in Indonesia for a long time, which presents a sustainable source for NCC production [24]. It also has high cellulose content, making it a promising candidate for future applications in drug delivery systems [25]. NCC-derived morphological, thermal, and mechanical properties of ramie crystallites—reinforced from ramie (*Boehmeria nivea* L. Gaud) (RNCC)—have been reported to successfully form nanocomposites with polyoxyethylene, starch, and polylactide [26,27,28]. Although previous studies have synthesized and used RNCC for various applications, its potential to enhance the solubility of poorly-soluble drugs has not yet been investigated. The main goal of this research is to improve the therapeutic effects and dissolution rate of the drug, furosemide. This study is in line with the growing need for eco-friendly drug delivery methods that take advantage of the renewable nature of ramie.

In this study, RNCC was obtained through the acid hydrolysis of α-cellulose using strong acid as a catalyst. Sulfuric acid (H_2_SO_4_) and hydrochloric acid (HCl) were used with varying concentrations and reaction times, as they are considered to be important parameters during acid hydrolysis [23]. H_2_SO_4_ was selected for its high success rate in producing NCC, while HCl has a low probability of leaving acid residues bound to NCC [29]. Moreover, it involves the formulation of furosemide/RNCC microspheres, utilizing polyvinyl alcohol (PVA) and sodium starch glycolate (SSG) as stabilizers and suspending agents, to enhance the solubility and stability of drug delivery systems [30]. PVA stabilizes suspensions by preventing aggregation due to its hydrophilic nature [30]. SSG acts by enhancing the ability of particles to disperse evenly in the formulation, as well as aiding in faster drug release [31,32]. These microspheres were evaluated for their physicochemical properties and ability to enhance furosemide solubility and bioavailability.

This research aims to contribute novel insights to the development of efficient drug delivery systems by focusing on the exploration of RNCC and its application in the formulation of furosemide/RNCC microspheres, which can improve the solubility and bioavailability of furosemide. The designated hydrolysis reaction conditions to synthesize high-quality RNCC and its incorporation into microspheres represent a potential breakthrough in addressing the solubility challenges associated with BCS class IV drugs, offering a sustainable and effective approach to enhance oral bioavailability.

## 2. Materials and Methods

### 2.1. Materials 

Plant materials were *Boehmeria nivea* L. Gaud plant, which was freshly collected from Wonosobo, Central Java, Indonesia, in April 2019. Taxonomic identification and authentication were conducted in the Department of Biology, Faculty of Mathematics and Natural Sciences, Padjadjaran University by Mr. Joko Kusmoro. Furosemide (Kimia Farma, Jakarta, Indonesia), acetic acid 0,1 N (Merck, Darmstadt, Germany), sodium hydroxide (NaOH, Merck, Darmstadt, Germany), sodium hypochlorite (NaClO, Merck, Darmstadt, Germany), hydrochloric acid (HCl, Merck, Darmstadt, Germany), sulfuric acid (H_2_SO_4_, Merck, Darmstadt, Germany) polyvinyl alcohol (PVA. Bratachem, Bandung, Indonesia), and sodium starch glycolate (SSG, Bratachem, Bandung, Indonesia) were used as received.

### 2.2. Preparation of RNCC

**Figure 1:**
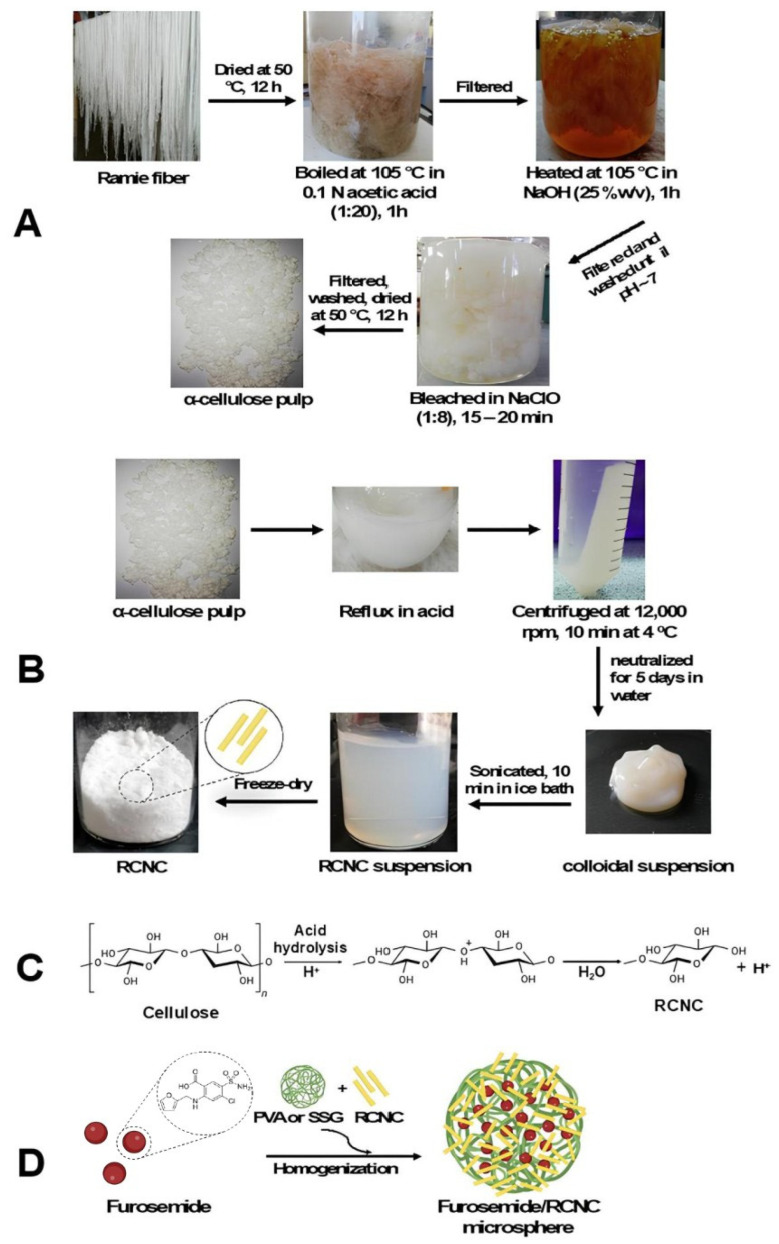
(**A**) Preparation of pulp, (**B**) acid hydrolysis of pulp to form RNCC, (**C**) its mechanism, and (**D**) schematic diagram of preparation of furosemide/RNCC microsphere.

Ramie (*Boehmeria nivea* L. Gaud) was washed and decorticated. Ramie fiber was washed and dried under the sun for 3 days. Then, it was cut into small pieces and dried in the oven at 40–50 °C for 12 h. As seen in Figure 1A, pre-hydrolysis was conducted by boiling it in acetic acid 0.1 N (1:20) at 105 °C for 1 h. After the residue was filtered and squeezed, it was heated with NaOH (25% *w*/*v*) at 105° C for 1 h, filtered, and washed until attaining a pH of 6–7. Further, it was bleached using NaClO 5% (1:8) for 15–20 min at room temperature. To form α-cellulose pulp, it was filtered, washed, and dried in the oven for 12–24 h at 50 °C.

To optimize α-cellulose hydrolysis catalysts, adjustments were made to the concentration and reaction time of HCl and H_2_SO_4_. A total of 100 mL acid was added to α-cellulose pulp (5 g) and refluxed at temperatures and reaction time, as shown in Table 1. After adding 100 mL of DI water, the mixture was cooled to room temperature. The suspension was centrifuged at 12,000 rpm for 10 min at a temperature of 4 °C. The obtained precipitate was then neutralized using a dialysis membrane (molecular weight cut-off (MWCO) 14 KDa; Sigma-Aldrich, St. Louis, MO, USA) for 5 days with DI water as the medium. After that, the colloidal suspension obtained was sonicated for 10 min in an ice bath and filtered with filter paper. The suspension was sonicated for 10 min before being dried with a freeze-dryer (Figure 1B).

### 2.3. Preparation of Furosemide/RNCC Microsphere

The furosemide/RNCC microsphere was made using different types of suspending agents, which were PVA and sodium starch glycolate (SSG). Furosemide/RNCC microspheres were formulated as shown in Table 2. It was prepared by dispersing RNCC into a PVA or SSG solution and stirring it at 2700 rpm for 30 min. Furosemide (1%) was then added to the RNCC suspension and mixed with a homogenizer at 9450 rpm (5 min) and at 5400 rpm (30 min) for PVA and SSG, respectively. The suspension was then stirred with a magnetic stirrer at 500 rpm for 4 h and filtered to obtain furosemide/RNCC microspheres (Figure 1D).

### 2.4. Characterization

#### 2.4.1. Particle Size Analyzer

The particle size distribution of RNCC and microsphere was determined using a Particle Size Analyzer (Beckman Coulter LS 13 320, Brea, CA, United States of America) at room temperature within the sub-micron scale range (40–2000 nm).

#### 2.4.2. Conductometric Titration

The conductometric titration method was used to quantify the sulfate density of RNCC. NaOH (0.005 N) was added drop-by-drop to the 0.5% RNCC suspension. The conductivity of the suspension versus the amount of aqueous NaOH solution was plotted. Sulfate density was calculated using Equation (1) [33]:

(1) $$Sulfate\;density=\frac{V\times N}{W}$$

where *V* is the consumption of NaOH (mL); *N* is the NaOH concentration; and *W* is the weight of RNCC.

#### 2.4.3. Transmission Electron Microscopy (TEM)

The morphology of RNCC was studied using a TEM (JEOL JEM-1400 series 120 kV (JEOL United States of America, Inc., Peabody, MA, USA) at a magnification of 5000–10,000 times and an accelerating voltage of 100 kV. A 10 μL drop of RNCC suspension was placed on copper grids coated with a carbon layer for examination.

#### 2.4.4. Fourier Transform Infrared (FT-IR) Spectroscopy

RNCC (1 mg) was ground with KBr (200–250 mg) to make a pellet. The functional group of RNCC was investigated by an FTIR spectrophotometer (Shimadzu, IR Prestige-21, Kyoto, Japan) at wavenumbers ranging from 500 to 4000 cm^−1^.

#### 2.4.5. X-Ray Diffractometry (XRD)

The X-ray diffraction pattern of RNCC was measured using an XRD (Rigaku Co., Ltd., Tokyo, Japan) with Cu-Kα radiation (λ = 1.5406 Å) at 40 kV and 30 mA. The measurements were taken in the range of 2θ = 5–60°. To calculate the crystallite size (*τ*), the Scherrer equation was used, as shown in Equation (2), where K (0.94) is the Scherrer constant, *β* is the width at the half maximum of the diffraction peak, and 2*θ* is the corresponding diffraction peak angle.

(2) $$\tau =\frac{K\lambda }{\beta \cos2\theta }\;$$

Additionally, the crystallinity index (CI) was calculated in Equation (3), where *I*_020_ is the intensity of the (020) lattice peak (2*θ*  =  22°) and the intensity attributed to amorphous (2*θ*  =  15°) [34].

(3) $$\mathrm{CI}=\frac{I_{020}-I_{am}}{I_{020}}\times 100$$

#### 2.4.6. Thermal Analysis Using Differential Scanning Calorimetry (DSC) and Thermogravimetric Analysis (TGA)

The thermal properties and thermal stability of RNCC were determined using DSC (Hitachi High-Tech Science Corporation; Tokyo, Japan) and TGA (HITACHI STA7300) respectively, at a temperature range of 30–600 °C and a heating rate of 10 °C/min. The sample in the form of a suspension was dried at room temperature. The DSC studies were performed by heating ~3–6 mg (solid) sample of each of the individual samples at a speed of 10 °C/min from 20 °C to 400 °C in a covered sample pan under nitrogen gas flow (60 mL/min).

### 2.5. Drug Loading and Encapsulation Efficiency

The drug loading on microspheres was determined using the spectrophotometer method. Furosemide (1 mg/mL) was diluted into 6, 7, 8, 9, and 10 ppm in methanol. Additionally, the microsphere (1 mg/mL) was dissolved in methanol, sonicated, and filtered. Furosemide content was determined using UV-Vis spectrophotometry at a wavelength of 271 nm [35]. The estimation of furosemide loading was then calculated using Equations (4) and (5) below [36]:

(4) $$\%\;drug\;loading=\frac{W_{t}}{W_{0}}\times 100$$

(5) $$\%\;encapsulation\;efficiency=\frac{l_{r}}{l_{t}}\times 100$$

where *W_t_* is the weight of the drug in the microsphere; *W_0_* is the weight of the microsphere; while *l_r_* is the actual loading and *l_t_* is the theoretical loading.

### 2.6. In Vitro Furosemide Dissolution in RNCC Microsphere

The in vitro dissolution study was conducted in a phosphate buffer medium (pH of 5.8) with a total volume of 900 mL. Microspheres, equivalent to 20 mg of furosemide, were placed into a dialysis bag of 12.000 kDa with 3 mL of dissolution media. The dialysis bag was securely attached to a paddle type II, and the dissolution process occurred at 37 °C with a stirring speed of 50 rpm. At specific intervals of 5, 10, 15, 30, 45, 60, 90, 120, and 150 min, a 5 mL sample of the solution was collected, and a fresh medium of the same volume was substituted. The concentration of the released drug in the dissolution medium was measured using a UV spectrophotometer [37].

## 3. Results

### 3.1. Characterization of RNCC

**Figure 2:**
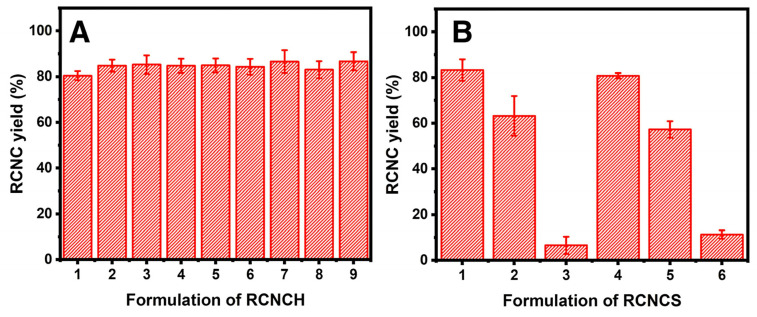
Percentage of RNCC yield obtained using the formulation of (**A**) RNCCH and (**B**) RNCCS.

RNCCH was synthesized under varying HCl concentrations and reaction times, with RNCCH 9 yielding the highest efficiency at 86.71 ± 4.00%. The yields of RNCCH 1 to RNCCH 8 ranged from 80.46 ± 1.98% to 86.59 ± 4.96%. In contrast, the synthesis and isolation of RNCCS resulted in more variable yields, ranging from as low as 6.60 ± 3.76% for RNCCS 3 to a maximum of 83.30 ± 4.72% for RNCCS 1, as shown in Figure 2.

Particle size analysis of the RNCC suspensions is summarized in Table 3. Among the RNCCH samples, the largest average particle size was observed in RNCCH 1 and RNCCH 6 (1.50 ± 0.57 nm), while the smallest was recorded in RNCCH 7 (1.35 ± 0.62 nm). For the RNCCS group, RNCCS 1 exhibited the highest mean particle size (1.49 ± 0.59 nm), whereas RNCCS 2, 5, and 6 showed the lowest value (0.12 nm). This study also included pH testing. RNCCH acidic pH levels ranged from 5.43 ± 0.05 to 6.76 ± 0.30. Meanwhile, in RNCCS, the acid pH level ranged from 3.19 ± 0.03 to 3.77 ± 0.07.

**Figure 3:**
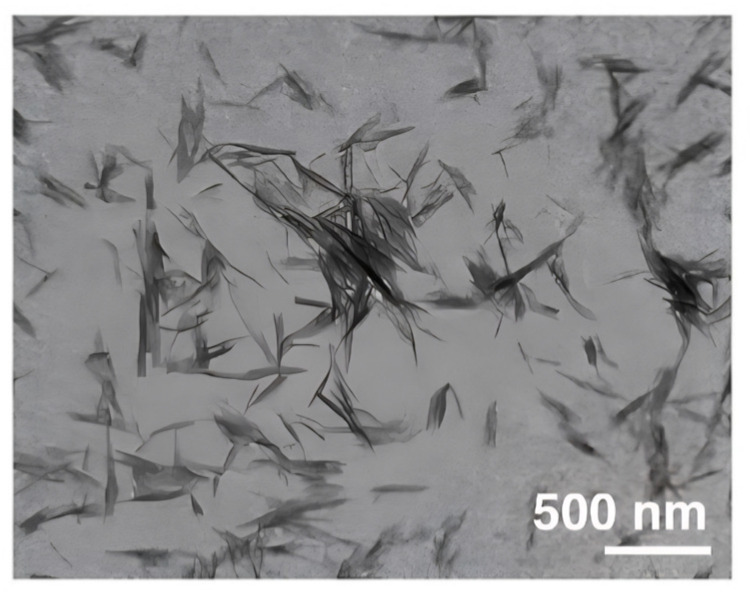
TEM image of RNCC.

**Figure 4:**
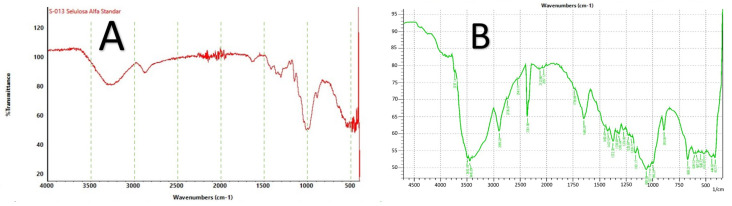
FTIR spectrum from alpha cellulose standard (**A**), ramie nanocrystalline cellulose (**B**).

**Figure 5:**
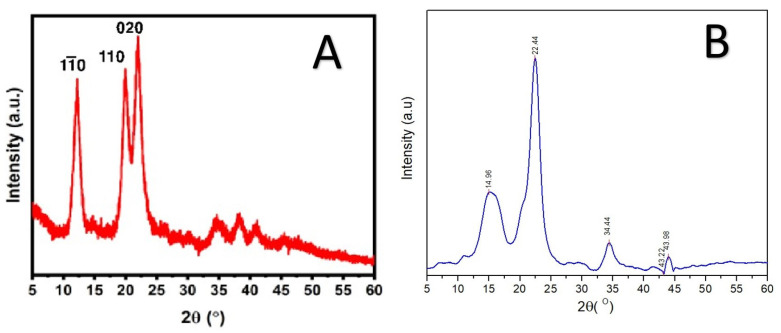
XRD diffractogram from ramie nanocrystalline cellulose (**A**), Avicel^®^ PH-101 (**B**).

The morphology of RNCC particles was examined using TEM, revealing a consistent needle-like crystalline shape, as shown in Figure 3. The FTIR spectra (Figure 4) displayed characteristic peaks corresponding to typical NCC features. Meanwhile, the XRD analysis presented in Figure 5 confirmed the crystalline structure of RNCC.

**Figure 6:**
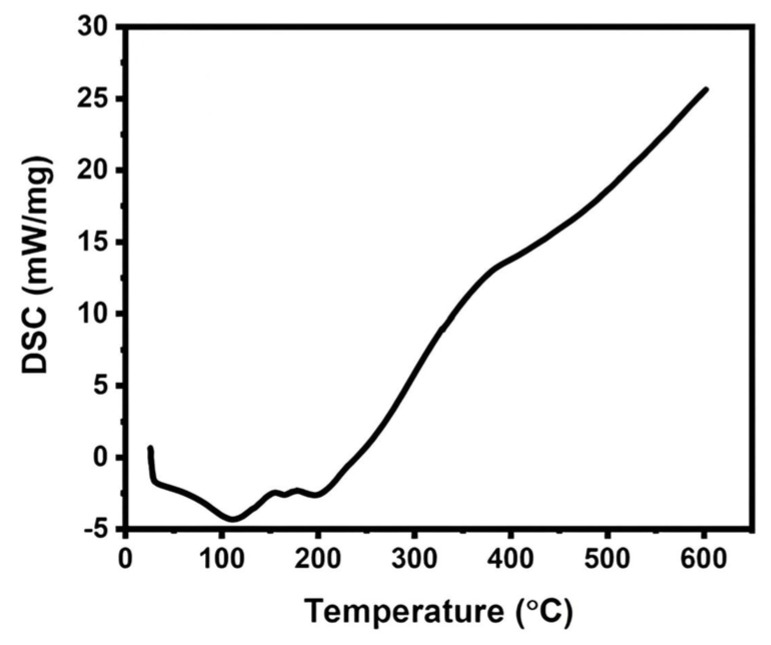
Thermal analysis of RNCC using DSC.

**Figure 7:**
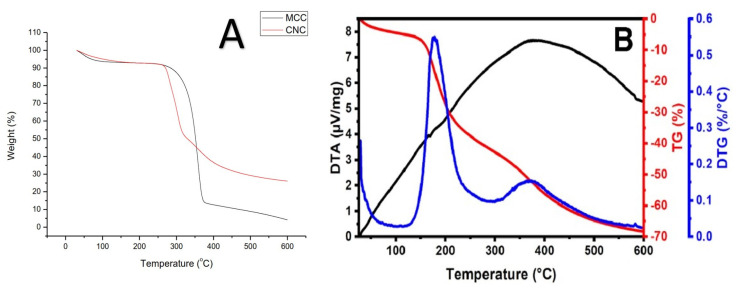
Thermal behavior of TGA from MCC and CNC from rice straw (**A**), TG-DTG from RNCC (**B**).

The thermal analysis of the isolated RNCC was conducted using DSC and TGA (Figure 6 and Figure 7). DSC analysis showed three endothermic peaks at 111 °C, 167 °C, and 198 °C, corresponding to water evaporation, depolymerization, and cellulose decomposition, respectively. TGA revealed three major weight loss phases, with a final residue of approximately 32% remaining at 600 °C

### 3.2. Characterization of Furosemide/RNCC Microsphere

Furosemide/RNCC microspheres were formulated by optimizing the stirring conditions. The particle size increased with higher RNCC and decreased with higher PVA (Table 4). Formulas with SSG showed size variation, with FS2 having the smallest (22.26 ± 15.68 μm) and FS1 the largest (36.11 ± 30.09 μm). The increased size compared to pure furosemide indicates RNCC attachment, though RNCC and SSG levels had minimal effect on the overall size.

**Figure 8:**
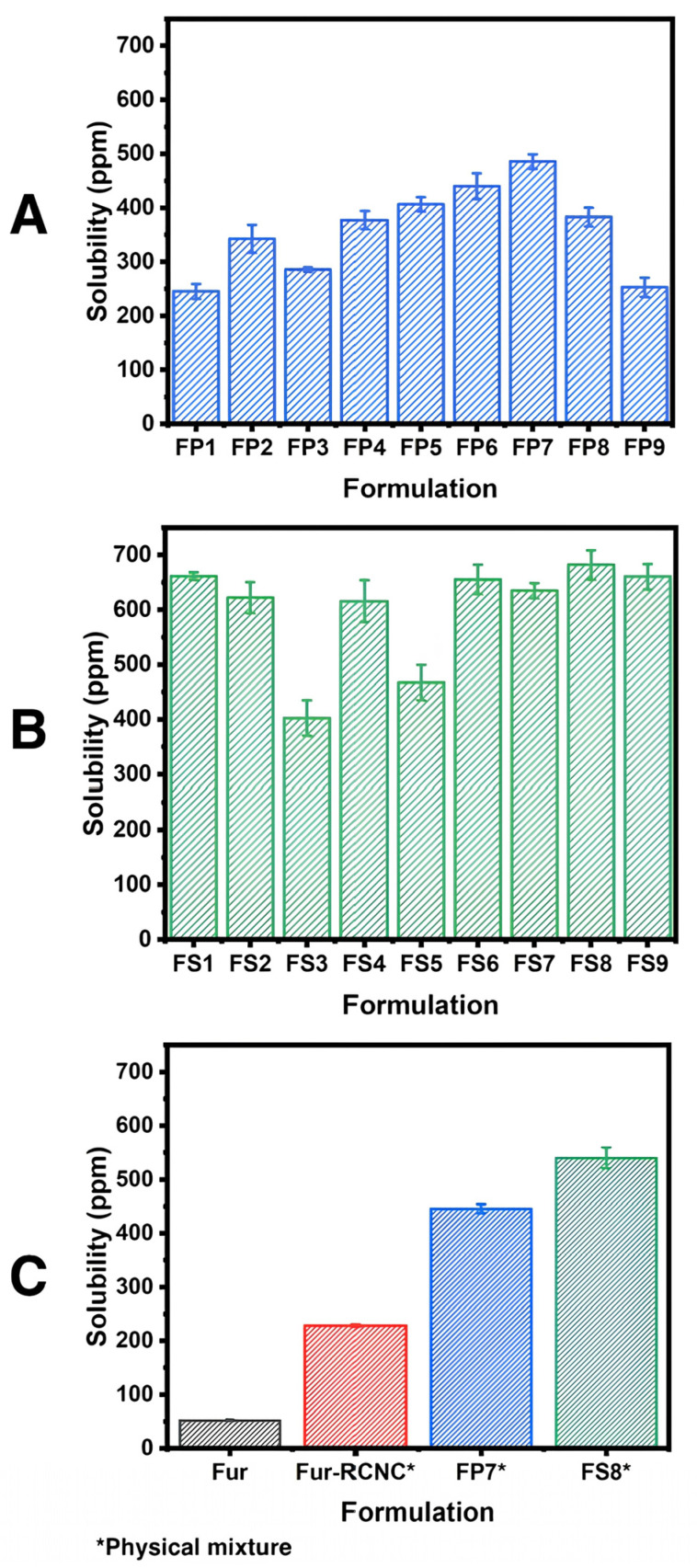
Solubility of furosemide/RNCC microsphere using the formulation of (**A**) FP and (**B**) FS. (**C**) Solubility of furosemide and physical mixture of furosemide and RNCC with and without the addition of suspending agent.

Figure 8A,B show that formulations FP7 (with PVA) and FS8 (with SSG) increased furosemide solubility by 9- and 12-fold compared to the pure drug (52.04 ppm).

### 3.3. Drug Loading and Encapsulation Efficiency of Furosemide

**Figure 9:**
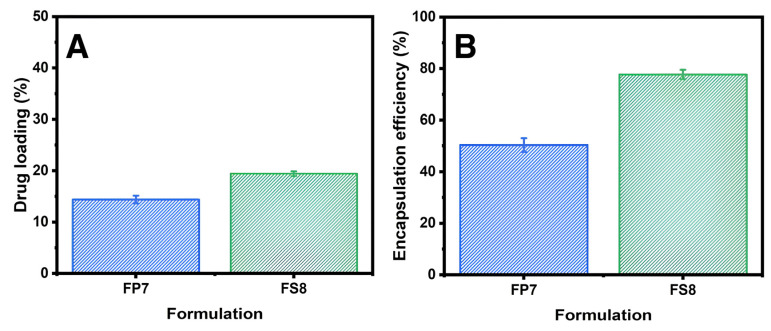
(**A**) Drug loading and (**B**) encapsulation efficiency of furosemide/RNCC microsphere.

Drug loading analysis of furosemide/RNCC microspheres (FP7 and FS8) showed a lower loading in FP7, likely due to PVA’s solubilizing effect on furosemide (Figure 9A). Encapsulation efficiency, shown in Figure 9B, was higher for FS8 than FP7, indicating that SSG more effectively binds furosemide than PVA, due to PVA’s hydrophilicity.

### 3.4. In Vitro Dissolution of Furosemide in RNCC Microspheres

**Figure 10:**
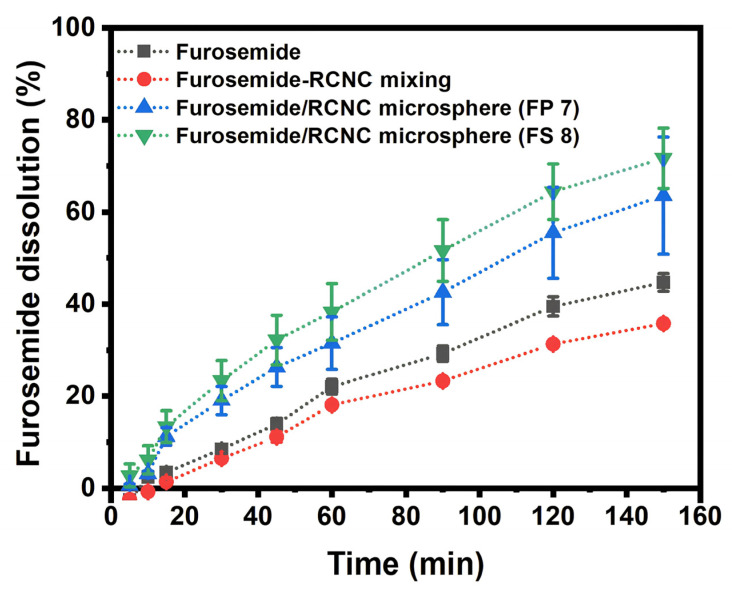
Dissolution of furosemide, furosemide-RNCC mixing, and furosemide/RNCC microsphere.

In vitro dissolution studies revealed that furosemide/RNCC microspheres (FP7 and FS8) exhibited significantly higher dissolution rates compared to pristine furosemide and the furosemide-RNCC physical mixture (1:1). After 150 min, FS8 showed the highest dissolution (71.7%), followed by FP7 (63.5%), furosemide (44.7%), and the furosemide-RNCC mixing (35.8%) (Figure 10). These findings suggest that the microsphere formulations enhance furosemide dissolution, with FS8 outperforming FP7, consistent with the saturated solubility test results.

## 4. Discussion

### 4.1. Characterization of RNCC

In the synthesis of RNCCH, varying HCl concentrations and reaction times were explored, with RNCCH 9 achieving the highest yield at 86.71 ± 4.00%. The yields for RNCCH 1 to RNCCH 8 range between 80.46 ± 1.98% and 86.59 ± 4.96%. Notably, these yields are lower than NCC isolated from MCC, attributed to the higher amorphous cellulose content in α-cellulose. Based on the two-way ANOVA statistical analysis of the RNCCH yield, it was found that changes in the hydrochloric acid concentration did not significantly affect the RNCCH yield (*p*-value 0.74). Similarly, the effect of reaction time on the NSRH yield was not significant (*p*-value 0.65), and there was no significant interaction between the hydrochloric acid concentration and hydrolysis reaction time (*p*-value 0.57). These findings suggest that neither the acid concentration, nor the reaction time, nor their interaction, significantly influenced the yields in this study. RNCCH synthesis results provided in Figure 2A indicate that longer reaction times generally lead to higher yields, except for a slight decrease using 20% HCl concentration for 6 h. While higher HCl concentrations enhance the RNCCH yield at a 2-h reaction time, prolonged hydrolysis negatively impacts the NCC yield, emphasizing the importance of optimizing reaction times for optimal yields and avoiding excessive hydrolysis to prevent cellulose degradation [38].

In the synthesis and isolation of RNCCS, varying yields were obtained, ranging from the lowest at 6.60 ± 3.76% for RNCCS 3 to the highest at 83.30 ± 4.72% for RNCCS 1. RNCCS 2 exhibited a higher standard deviation, possibly due to its lengthy process, posing a risk of mass loss during decantation in the neutralization process. The two-way ANOVA analysis revealed that the yield of RNCCS was significantly influenced by the sulfuric acid concentration (*p*-value 0.00). However, the reaction time did not have a significant effect on the yield (*p*-value = 0.66), nor did the interaction between sulfuric acid concentration and reaction time (*p*-value = 0.33). These results indicate that sulfuric acid concentration plays a crucial role in determining the RNCCS yield, while reaction time and their interaction do not have a significant impact. Figure 2B indicates that an increase in H_2_SO_4_ concentration at the same reaction time results in a decrease in RNCCS yield. It is attributed to an accelerated cellulose hydrolysis rate, leading to increased soluble amorphous cellulose and smaller crystalline cellulose particles. This phenomenon is evident in the RNCCS suspension after neutralization, appearing more transparent due to gel formation. NaOH neutralization was avoided to prevent the formation of sodium sulfate salt crystals, which would complicate the RNCCS isolation process [39].

Particle size analysis was performed on the RNCC suspension (Table 3). In RNCCH, the highest mean particle size was found in RNCCH 1 and 6 at 1.50 ± 0.57 nm, while the lowest was in RNCCH 7 at 1.35 ± 0.62 nm. The two-way ANOVA analysis indicates that the increase in hydrochloric acid concentration and reaction time did not significantly influence the particle size of the RNCCH formed, with *p*-values of 0.44 and 0.45, respectively. This suggests that neither the hydrochloric acid concentration, nor the reaction time, nor their interaction, has a significant effect on the particle size of the RNCCH under the tested conditions. In contrast to the previous findings [40], it was observed that the reduction in particle size during RNCC synthesis was not affected by the concentration of HCl (15–25%) or the reaction time (2–6 h) due to particle instability caused by sedimentation from RNCCH particle flocculation [38]. Moreover, the wide particle size distribution of RNCCH, with high standard deviations within the micron size range, suggests that the hydrolysis reaction may not be optimal.

The hydrolysis using HCl did not yield particles within the expected nanometer range characteristic of NCC, indicating its limited efficiency in breaking down amorphous regions of cellulose [41]. Although HCl was able to partially hydrolyze the cellulose structure, it produced relatively larger particles, likely due to insufficient depolymerization and a less controlled reaction environment compared to H_2_SO_4_ [38]. Sulfuric acid has been widely reported to facilitate more effective cellulose breakdown, resulting in finer, more crystalline NCC particles [38]. In contrast, HCl-treated samples not only exhibited larger and more polydisperse particles (as shown in Table 3), but also demonstrated lower sulfate densities, indicating limited surface functionalization [33]. The lack of sufficient sulfate group incorporation reduces electrostatic repulsion, which is essential for stabilizing smaller colloidal nanocrystals in suspension [42]. Therefore, HCl hydrolysis is less suitable for producing high-quality NCC compared to H_2_SO_4_-based methods.

Table 3 indicates that at a 2 h reaction time, a higher HCl concentration leads to a lower RNCCH pH. The influence of reaction time on the pH variation decreases at a 3 h reaction time before increasing at 6 h, possibly indicating chloride residue formation with cellulose hydroxyl groups. Despite this, the tendency towards a neutral pH demonstrates effective acid neutralization with a dialysis membrane, resulting in neutral or uncharged RNCCH particles that are easy to flocculate or difficulties in forming a colloidal suspension in water [43].

On the other hand, Table 3 shows the relationship between the influence of H_2_SO_4_ concentration and reaction time on RNCCS particle size. The measured particle sizes showed that at 45% H_2_SO_4_ concentration, two peaks in the particle size graph indicated incomplete hydrolysis, leaving micrometer-sized cellulose particles. Higher sulfuric acid concentrations resulted in smaller RNCCS particles, but differences were insignificant between 50% and 55%. The two-way ANOVA analysis reveals that sulfuric acid concentration significantly affects the particle size of the resulting RNCCS (*p*-value = 0.01). This indicates that changes in sulfuric acid concentration have a notable impact on the particle size of RNCCS, while other factors may not show significant effects. Conversely, the difference in reaction time is unlikely to affect RNCCS particle size significantly. This study also included the pH testing of RNCCS, revealing acidic pH levels ranging from 3.19 ± 0.03 to 3.72 ± 0.06. The reaction time (45 and 60 min) did not significantly affect the particle size of RNCCS (*p*-value = 0.6). This indicates that changes in reaction time within this range do not have a notable impact on the particle size of RNCCS. Higher H_2_SO_4_ concentrations and longer hydrolysis reaction times resulted in lower RNCCS pH. However, RNCCS at 55% H_2_SO_4_ concentration showed a higher pH, potentially due to the low RNCCS content in the suspension and very low product yield. The acidic pH of RNCCS could be attributed to sulfate ions penetrating the cellulose hydroxyl side, considering the neutralization process with dialysis membranes was conducted until the demineralized water reached a neutral state.

Therefore, an analysis of the sulfate density penetrating RNCC particles is essential. This study conducted sulfate density analysis on RNCC particles, excluding RNCCS 3 and 6 due to low yield. Sulfate density testing involved conductometric titration, revealing that higher H_2_SO_4_ concentrations and longer reaction times significantly increased sulfate density. The results are consistent with the previous research indicating that increased acid concentration and reaction time enhance hydrolysis reaction strength, resulting in NCC with smaller particle sizes and higher yields but also higher sulfate levels [43]. RNCCS 2 with 50% sulfuric acid concentration at 45 °C for 45 min was chosen for further characterization due to its nanoparticle size range and lower sulfate density compared to RNCCS 5.

The morphology of RNCC particles was examined using TEM, and Figure 3 revealed a consistent needle-like crystal shape. The optimized RNCC exhibited uniform size, shape, and characteristics typical of nanocrystalline cellulose. The morphology showed homogeneity without the presence of MCC particles. Additionally, the arrangement of RNCC in the suspension indicated the absence of flocculation or particle aggregation, attributed to repulsive forces between particles due to sulfate groups on the RNCC surfaces [44].

The FTIR spectra indicated typical NCC features, as shown in Figure 4. Notable peaks in the range of 3500–3300 cm^–1^ included those representing O–H stretching vibrations, which arise from both intra- and intermolecular hydrogen bonding within the cellulose group [42,45]. Moreover, bands at around 1420–1059 cm^−1^ are typical of the intermolecular hydrogen at the C_6_ (aromatic group) of cellulose [46] and can be assigned in the FT-IR spectra of RNCC also (Figure 4). Generally, a band at 1161 is assigned as C–O–C stretching vibrations and 893 cm^−1^ is associated to C–H rock vibration of the characteristic β (1 → 4)-glycosidic linkage [47]. In RNCC, a sharp peak was observed at 1648 cm^−1^ which was different from the standard (alpha cellulose), the peak was the angular O–H bending of water molecules as a result of cellulose-water interactions [48], but it could also be the presence of the aromatic C = C stretch of aromatic rings in the lignin [49].

The XRD analysis depicted in Figure 5 shows the crystallinity of synthesized RNCC, showing three sharp peaks at 12.11°, 19.85°, and 22.03°, corresponding to (1–10), (110), and (020) planes, consistent with typical cellulose II crystal patterns [34]. The crystallite size calculated using the Scherrer equation was 0.93 nm, and the crystallinity index was determined to be 96.22%. This value indicates a highly ordered crystalline structure and demonstrates the effective removal of amorphous regions from α-cellulose during the hydrolysis process. When compared to commercial microcrystalline cellulose (MCC), such as Avicel^®^ PH-101, which typically exhibits a crystallinity index of approximately 56.68%, the RNCC synthesized in this study shows a markedly superior degree of crystallinity. This substantial increase aligns with the expected structural refinement of nanocrystalline cellulose and is indicative of successful synthesis conditions. Moreover, the crystallinity index obtained in this work is higher than those reported in previous studies, which documented values around 77.80% for cellulose nanocrystals derived from similar biomass sources [42]. The high crystallinity not only confirms the effective elimination of amorphous components but also suggests enhanced thermal and mechanical stability of the RNCC. This is advantageous for its application in drug delivery systems, where crystallinity contributes to structural integrity, suspension stability, and overall performance of the nanocarrier matrix.

Ramie-derived cellulose nanocrystals (CNCs) exhibit high crystallinity (96.2%) and needle-like morphology, which are favorable characteristics for drug delivery. Compared to other sources, such as jackfruit peel [19] or seaweed [21], ramie offers higher cellulose content and mechanical strength, resulting in CNCs with more uniform particle morphology and better colloidal stability. Additionally, ramie is abundantly cultivated in Indonesia, supporting sustainability and large-scale production [21,24].

The thermal analysis of isolated RNCC was conducted using DSC and TGA (Figure 6 and Figure 7). In DSC, three endothermic phenomena were observed at 111 °C, 167 °C, and 198 °C, representing water evaporation, depolymerization, and cellulose decomposition, respectively. TGA revealed three mass reduction phases, including water loss up to 100 °C, significant mass reduction at 181.9 °C due to cellulose depolymerization accelerated by sulfate residues, and mass reduction at 369.6 °C indicating cellulose decomposition. The remaining mass after 600 °C was around 32%, representing the ash mass undergoing oxidation [38,42]. The thermal properties of RNCC depict typical thermal properties of nanocrystalline cellulose with relatively good thermal stability up to around 180 °C. Thus, the characterization analysis confirmed that the isolated RNCC possesses solid characteristics similar to those of previous studies but with a higher crystallinity index

Many studies in the literature have discussed that nanocrystalline cellulose usually has lower thermal stability than microcrystalline, as in the comparison in Figure 7 obtained from rice straw. Figure 7A shows the TGA curves of MCC (as reference standard) and CNC. The initial weight loss of both MCC and CNC occurs in the range of 30 °C to 120 °C, which can be attributed to water evaporation in the sample due to their inherent hydrophilic nature. The second stage of weight loss (289 °C–380 °C) can be ascribed to the cellulose depolymerization, generating volatile hydrocarbons and CO_2_ gases. The onset degradation temperature of MCC which is at 315 °C is higher when compared to 289 °C of CNC. Additionally, the maximum decomposition temperature of MCC at 355 °C was found to be significantly higher than CNC at 300 °C. It is possible that the increase in surface area of CNC might be the contributing factor in its diminished thermal stability as more surface is exposed to heat which accelerated the degradation process [50]. Likewise, the same pattern was obtained in the RNCC that was produced from this study. Where three phases of RNCC mass reduction are obtained, first, it represents a water loss of less than 10% of the RNCC mass up to a temperature range of 100 °C. Second, a significant mass decrease of up to 40% by mass at 281.9 °C reflects the accelerated cellulose depolymerization in the presence of sulfate residues on the surface of RNCC particles, as well as a decrease in mass in the temperature range of 369.6 °C which describes the decomposition of cellulose into gas products with a low molecular weight. The weight of the RNCC remaining after 600 °C was approximately 32% of the initial mass of the sample tested.

### 4.2. Characterization of Furosemide/RNCC Microsphere

The formulation process of furosemide/RNCC microspheres involves optimizing the stirring speed and duration. The suspending agent is used to facilitate the interaction between RNCC and the drug molecule during homogenization, resulting in the adherence of RNCC to the drug molecule [32]. Particle size analysis indicates that increasing the RNCC concentration in the microsphere suspension leads to larger particle sizes, while higher PVA concentration tends to decrease the particle size (Table 4). Microspheres with SSG in suspension show varying particle sizes, with formula FS2 exhibiting the smallest (22.26 ± 15.68 μm) and FS1 the largest (36.11 ± 30.09 μm). The larger size of furosemide/RNCC microspheres compared to pristine furosemide suggests the successful attachment of RNCC on furosemide particles with SSG. However, variations in RNCC and SSG concentrations were negligible to the size of the microspheres.

In Figure 8A,B, the solubility test of furosemide/RNCC microspheres in water reveals that the use of PVA with FP7 formulation and SSG with FS8 formulation significantly increased solubility. The solubility is up to 9 times and 12 times higher than pristine furosemide (52.04 ppm). Statistically, the increase in NSR and PVA concentrations did not significantly affect the saturated solubility of furosemide in the microspheres (*p*-values of 0.75 and 0.33). Similarly, the increase in NSR and SSG concentrations did not significantly affect the saturated solubility of furosemide in the microspheres (*p*-values of 0.789 and 0.589). Polyvinyl alcohol (PVA) is known for its hydrophilic nature, film-forming capability, and ability to improve drug wettability and dispersion, which can increase the surface area available for solubilization [51]. Similarly, sodium starch glycolate (SSG) acts as a super-disintegrant with high water uptake, facilitating rapid swelling and increasing the exposure of drug particles to the dissolution medium, enhancing solubility [52]. The solubility enhancement achieved (9–12×) using RNCC-based microspheres exceeds that of some conventional methods. For instance, solid dispersions typically yield 2–5× increases [12], while nanosizing techniques may improve solubility by 3–10× depending on the drug and excipient compatibility [8]. Therefore, the observed enhancement using ramie-derived CNC microspheres is comparable or superior, especially considering the biocompatibility, renewability, and eco-friendly processing of RNCC. However, increasing the concentration of RNCC, PVA, or SSG only has a slight impact on furosemide solubility in microspheres. Furthermore, the saturation solubility of the physical mixture of furosemide and RNCC (Fur/RNCC-mixing) at a 1:1 ratio and the physical mixture of Formula FP7 (FP7-mixing) and Formula FS8 (FS8-mixing) were also tested. Figure 8 shows that FS8-mixing achieves the highest solubility. Even just the mixing of furosemide with RNCC increases solubility 4 times higher than pristine furosemide. Additionally, the addition of a suspending agent to the mixture further increased the solubility up to 8 times with PVA and 10 times with SSG. This emphasizes that the formation of furosemide/RNCC microspheres has better solubility compared to physical mixtures. FP7 microspheres have a solubility of 485.98 ppm, whereas the FP7-mixing has a solubility of 445.37 ppm. A similar trend was observed in the FS formula, where microspheres have a solubility of 644.06 ppm compared to 540.12 ppm in their physical mixture.

### 4.3. Drug Loading and Encapsulation Efficiency of Furosemide

The observation of drug loading describes the amount of furosemide content in microspheres. The microspheres subjected to this test are the selected formula, which is furosemide/RNCC microspheres on FP7 and FS8. Figure 9A illustrates that the drug loading in furosemide/RNCC microspheres of formula FP7 was lower than that of formula FS8. This may be attributed to the presence of PVA in FP7, which is water-soluble and enhances the solubility of furosemide in water as a dispersant during the manufacturing process. As a result, furosemide partially dissolved and passed through the filtration. Furthermore, the encapsulation efficiency in Figure 9B illustrates the ability of suspending agents to bind with furosemide into the furosemide/RNCC microsphere system. The encapsulation efficiency data appear proportional to drug loading, whereas furosemide/RNCC microspheres FS8 have higher furosemide encapsulation efficiency than FP7. This data indicates that SSG has a better ability to bind furosemide than PVA due to the hydrophilicity of PVA, which causes difficulty in interacting with the furosemide [53].

### 4.4. In Vitro Dissolution of Furosemide in RNCC Microspheres

This study involved the in vitro dissolution analysis of various samples, including pristine furosemide, furosemide-RNCC mixing (1:1), and furosemide/RNCC microspheres formula FP7 and FS8. Statistical analysis was conducted using one-way ANOVA followed by Tukey’s post-hoc test to evaluate the differences in furosemide dissolution among four formulations. ANOVA results indicated statistically significant differences (*p* < 0.05) at all observed time points from 5 to 120 min, confirming that the formulation type significantly influenced dissolution behavior.

Tukey’s HSD test revealed that FS8 consistently outperformed both the pristine furosemide and furosemide-RNCC mixing (1:1) from as early as minute 5 (*p* < 0.01), with significant improvements also observed at 10, 15, 30, 45, 60, 90, and 120 min. The FP7 microsphere also showed significantly enhanced dissolution compared to the pure drug and physical mixture at several time points, notably after minute 15 (*p* < 0.05). While FS8 often exhibited slightly higher mean dissolution values than FP7, their difference was not statistically significant across all time points (*p* > 0.05), suggesting comparable performance between the two microsphere systems. These findings confirm that the incorporation of RNCC into microsphere formulations, especially FS8 using sodium starch glycolate, substantially improves the dissolution rate of furosemide, which may enhance its bioavailability. The effect was not only immediate but also sustained through the duration of the dissolution period studied.

The baseline curve, established using dissolution media, followed a linear regression equation y = 0.0522 × + 0.0595. Figure 10 shows the slow dissolution of furosemide in the phosphate buffer, where furosemide was first observed at 10 min and continuously increased to reach 44.7% dissolution after 150 min. The furosemide-RNCC mixing (1:1) showed that furosemide began to be observed in the dissolution media after 15 min and steadily increased to reach 35.8% after 150 min. It exhibits lower dissolution than pristine furosemide, which differs from the result of water solubility analysis due to the formation of a viscous gel-like barrier by RNCC, which hinders drug diffusion through the dialysis membrane. The limited permeability of the membrane to larger colloidal structures may also contribute to this observation [37,52]. NCC tends to swell and form a liquid crystal-like network when dispersed in aqueous media. This network can obstruct drug diffusion through the dialysis membrane by creating a physical hindrance or increasing the local viscosity near the membrane interface. Additionally, dialysis membranes with molecular weight cut-offs (MWCOs) such as 12,000 Da may not be optimally permeable to drug-NCC complexes or larger aggregates, especially when NCC interacts with the drug or forms a gel layer [37,53]. Dissolution testing of furosemide/RNCC microspheres FP7 and FS8 with PVA and SSG suspension revealed the furosemide presence after 5 min and reached 63.5% and 71.7% after 150 min. According to the results, the dissolution rates in the gastrointestinal tract were the lowest in the furosemide-RNCC mixing, followed by the pristine furosemide, FP7 microspheres, and the highest in FS8 microspheres. This suggests that the microspheres have a higher dissolution rate compared to pristine furosemide and its physical mixture with RNCC. Additionally, FP7 microspheres showed a lower dissolution rate than FS8, which is consistent with patterns from saturated solubility tests. The presence of the suspending agent and the microsphere reservoir system supports these findings.

## 5. Conclusions

RNCC was successfully isolated using H_2_SO_4_ as a catalyst for hydrolysis, resulting in particles with specific characteristics, including a size of 120 nm and a sulfate density of 133.09 mmol/kg. The RNCC exhibited needle-slike particle morphology, distinctive functional groups, high crystallinity of 96.2%, and good thermal stability. HCl, as an alternative catalyst, failed to produce NCC due to the resulting particle size being outside the desired nanoscale range. The optimization of cellulose hydrolysis conditions demonstrated that the H_2_SO_4_ concentration significantly influenced RNCC yield, particle size, pH, and sulfate density. The research extended the application of RNCC to improve the solubility of a class IV BCS drug, furosemide, in microsphere form. Using PVA and sodium starch glycolate SSG as suspending agents, the microspheres exhibited increased solubility and dissolution of furosemide when incorporating RNCC. The choice of the suspending agent also played an important role, with SSG outperforming PVA in enhancing furosemide solubility. In conclusion, RNCC showed promise in enhancing the solubility of class IV BCS drugs in microsphere formulations. Future research can explore the use of RNCC with different types of drugs, evaluate its effectiveness through animal or clinical testing, and develop RNCC-based drug delivery systems on a larger scale. This will support the development of more sustainable and environmentally friendly pharmaceutical products.

## Figures and Tables

**Table 1:** Synthesis of RNCC with various acid concentrations and reaction times.

Formulation	Acid	Concentration (%)	Temperature (°C)	Time (min)
RNCCH 1	HCl	15	60	120
RNCCH 2	HCl	20	60	120
RNCCH 3	HCl	25	60	120
RNCCH 4	HCl	15	60	180
RNCCH 5	HCl	20	60	180
RNCCH 6	HCl	25	60	180
RNCCH 7	HCl	15	60	360
RNCCH 8	HCl	20	60	360
RNCCH 9	HCl	25	60	360
RNCCS 1	H_2_SO_4_	45	45	45
RNCCS 2	H_2_SO_4_	50	45	45
RNCCS 3	H_2_SO_4_	55	45	45
RNCCS 4	H_2_SO_4_	45	45	60
RNCCS 5	H_2_SO_4_	50	45	60
RNCCS 6	H_2_SO_4_	55	45	60

RNCCH: ramie cellulose nanocrystal using HCl; RNCCS: ramie cellulose nanocrystal using H_2_SO_4_.

**Table 2:** Optimization of RNCC and suspending agent concentration in furosemide/RNCC microsphere formulation.

Materials	FP1 (g)	FP2 (g)	FP3 (g)	FP4 (g)	FP5 (g)	FP6 (g)	FP7 (g)	FP8 (g)	FP9 (g)
Furosemide	1	1	1	1	1	1	1	1	1
RNCC	0.5	1	2	0.5	1	2	0.5	1	2
PVA	0.5	0.5	0.5	1	1	1	2	2	2
	**FS1 (g)**	**FS2 (g)**	**FS3 (g)**	**FS4 (g)**	**FS5 (g)**	**FS6 (g)**	**FS7 (g)**	**FS8 (g)**	**FS9 (g)**
Furosemide	1	1	1	1	1	1	1	1	1
RNCC	0.5	1	2	0.5	1	2	0.5	1	2
SSG	0.25	0.25	0.25	0.5	0.5	0.5	0.5	0.5	1

FP: formulation using PVA; FS: formulation using SSG.

**Table 3:** Particle size analysis and pH of RNCC (*n* = 3).

Sample	Particle Size (nm)	pH	Sulfate Density (mmol/kg)
RNCCH 1	1500 ± 0.56	6.76 ± 0.30	-
RNCCH 2	1410 ± 0.59	6.72 ± 0.20	-
RNCCH 3	1410 ± 0.60	6.25 ± 0.14	-
RNCCH 4	1420 ± 0.59	6.11 ± 0.05	-
RNCCH 5	1410 ± 0.58	6.13 ± 0.03	-
RNCCH 6	1500 ± 0.57	6.18 ± 0.03	-
RNCCH 7	1350 ± 0.62	6.29 ± 0.01	-
RNCCH 8	1380 ± 0.60	6.39 ± 0.03	-
RNCCH 9	1450 ± 0.58	5.43 ± 0.05	-
RNCCS 1	1490 ± 0.59	3.31 ± 0.08	85.44 ± 15.39
RNCCS 2	120 ± 0.21	3.28 ± 0.03	134.69 ± 2.39
RNCCS 3	340 ± 0.65	3.77 ± 0.07	-
RNCCS 4	1400 ± 0.59	3.23 ± 0.03	66.08 ± 29.90
RNCCS 5	120 ± 0.17	3.19 ± 0.03	154.02 ± 1.85
RNCCS 6	120 ± 0.16	3.72 ± 0.06	-

**Table 4:** Particle size analysis of furosemide/RNCC microsphere (data are mean ± SD, n = 3).

Sample	Particle Size (μm)	
	FP *	FS *
1	8.77 ± 8.72	36.11 ± 30.09
2	6.47 ± 6.51	22.26 ± 15.68
3	14.15 ± 14.94	27.73 ± 18.89
4	8.74 ± 9.81	31.12 ± 24.70
5	8.74 ± 9.81	31.10 ± 23.70
6	9.31 ± 10.52	29.80 ± 32.02
7	6.51 ± 7.20	23.14 ± 17.53
8	6.66 ± 7.80	32.82 ± 19.52
9	6.14 ± 6.39	33.36 ± 18.28

* FP: formulation using PVA; FS: formulation using SSG.

## Data Availability

The data are contained within the article.

## Abbreviations

The following abbreviations are used in this manuscript:

RNCC	Ramie Cellulose Nanocrystals
BCS	Biopharmaceutical Classification System
MCC	Microcrystalline Cellulose
NCC	Nanocrystalline Cellulose
H_2_SO_4_	Sulfuric Acid
HCl	Hydrochloric Acid
PVA	Polyvinyl Alcohol
NaOH	Sodium Hydroxide
NaClO	Sodium Hypochlorite
SSG	Sodium Starch Glycolate
TEM	Transmission Electron Microscopy
FT-IR	Fourier Transform Infrared
XRD	X-Ray Diffractometry
DSC	Differential Scanning Calorimetry
TGA	Thermogravimetric Analysis
